# Quasi-Monopolar Stimulation: A Novel Electrode Design Configuration for Performance Optimization of a Retinal Neuroprosthesis

**DOI:** 10.1371/journal.pone.0073130

**Published:** 2013-08-26

**Authors:** Gita Khalili Moghadam, Robert Wilke, Gregg J. Suaning, Nigel H. Lovell, Socrates Dokos

**Affiliations:** Graduate School of Biomedical Engineering, University of New South Wales, Sydney, New South Wales, Australia; University of Regensburg, Germany

## Abstract

In retinal neuroprostheses, spatial interaction between electric fields from various electrodes – electric crosstalk – may occur in multielectrode arrays during simultaneous stimulation of the retina. Depending on the electrode design and placement, this crosstalk can either enhance or degrade the functional characteristics of a visual prosthesis. To optimize the device performance, a balance must be satisfied between the constructive interference of crosstalk on dynamic range and power consumption and its negative effect on artificial visual acuity. In the present computational modeling study, we have examined the trade-off in these positive and negative effects using a range of currently available electrode array configurations, compared to a recently proposed stimulation strategy – the quasi monopolar (QMP) configuration – in which the return current is shared between local bipolar guards and a distant monopolar electrode. We evaluate the performance of the QMP configuration with respect to the implantation site and electrode geometry parameters. Our simulation results demonstrate that the beneficial effects of QMP are only significant at electrode-to-cell distances greater than the electrode dimensions. Possessing a relatively lower activation threshold, QMP was found to be superior to the bipolar configuration in terms of providing a relatively higher visual acuity. However, the threshold for QMP was more sensitive to the topological location of the electrode in the array, which may need to be considered when programming the manner in which electrode are simultaneously activated. This drawback can be offset with a wider dynamic range and lower power consumption of QMP. Furthermore, the ratio of monopolar return current to total return can be used to adjust the functional performance of QMP for a given implantation site and electrode parameters. We conclude that the QMP configuration can be used to improve visual information-to-stimulation mapping in a visual prosthesis, while maintaining low power consumption.

## Introduction

A retinal neuroprosthesis employs an implanted multielectrode array to induce artificial vision in the visually impaired [1], [2]. The most effective stimulation strategy to map spatiotemporal visual information with a multielectrode array is via parallel stimulation of the electrodes [3], [4], under the assumption of independent electrode performance. However, the independence of the electrodes to elicit distinct and punctate phosphenes during concurrent stimulation is limited by electric crosstalk, namely the spatial interaction between their individual electric field profiles [3], [5]–[11]. Crosstalk possesses both advantages and disadvantages that will be described below.

Electric crosstalk has a constructive effect on the activation threshold of each electrode during concurrent stimulation. McCreery et al. [11] showed that crosstalk between simultaneously stimulated monopolar (MP) electrodes could boost the effect of field summation, leading to a decrease in activation threshold for each electrode. For a given electrode, the difference in threshold between single and multielectrode stimulation is defined as the threshold shift for that electrode. A greater threshold shift corresponds to a higher degree of crosstalk. There may be several advantages to this threshold shift. One major advantage is an increasing dynamic range of stimulation, defined as the difference between activation threshold current and the maximum safe current that can be applied before the electrode charge injection limit is exceeded. Another advantage is that threshold shift due to the superposition of the field profiles will decrease overall stimulus current and thus the power consumption of the device, thereby increasing the time needed before having to recharge the battery that powers the implant.

Despite the constructive interference of crosstalk on activation threshold, crosstalk also has a negative effect on artificial visual acuity. For artificial vision, visual acuity may be understood as the spatial frequency of stimulation with a high-contrast square-wave grating – bright and dark bars [8]. The spatial frequency of such a grating is directly related to the density of pixels (the pitch of the electrode array), which can be assessed by the ability of subjects to discriminate one stimulation site from its neighbors. It can be hypothesized that increasing the density of active electrodes would enhance the information content of the perceived image, thus providing high-acuity perception. However, the crosstalk effect is a physical constraint that limits artificial visual acuity. The disadvantage is that crosstalk leads to the undesirable activation of the target retinal cells in the overlap of the fields, consequently impairing the contrast of grating stimulation [5], [8].

The aim of the present study was to develop a strategy for designing an electrode array capable of balancing the constructive and destructive interferences of crosstalk. For visual prostheses employing multielectrode arrays, the electrode configuration contributes significantly to the targeted effect of stimulation. It contributes to spread of electric field through the retina and thus determines spatial selectivity of retinal ganglion cell (RGC) activation. Conventional retinal implants predominantly operate in MP mode, whereby current is returned via a distant return electrode. MP stimulation provides a broad spatial spread of the electric fields, producing an overlap of activation profiles which ultimately leads to a degradation of artificial visual acuity [2], [8], [12], [13] while increasing the threshold shift. To improve visual acuity by confining the fields, one crosstalk reduction technique is to utilize local return electrodes, resulting in the restriction of current flow between adjacent active and return electrodes. One example of focused electrical stimulation is the hexagonally-guarded electrode configuration, whereby each “hex” consists of one disk-shaped active electrode and six disk-shaped return electrodes, each returning one sixth of the active current [14].

Computational modeling studies [8], [15] have suggested that the hexagonal configuration evokes relatively restricted spatial retinal activation. Consequently, the contrast of retinal activation patterns is higher with hexagonally-arranged return electrodes than MP stimulation at an equal current level [8]. This implies that the hexagonal configuration is less efficient in terms of threshold shift. Further, the hexagonal configuration has two significant drawbacks. One is the large size of the electrode unit per pixel, which results in a larger distance between pixels. Accordingly, stimulation using multiple electrodes per pixel – for example, seven electrodes with the hexagonal configuration – impairs spatial sampling frequency and eventually results in a loss of artificial visual acuity. In contrast, stimulation with the MP configuration has the advantage of providing higher artificial visual acuity [8]. Yet, superposition of the broad field profiles with MP stimulation, particularly at large distances from the electrode array, results in a low contrast grating stimulation and consequently impairs artificial visual acuity. Thus, high spatial resolution MP stimulation has only limited clinical benefit [7]. A second disadvantage of the hexagonal configuration is that the activation threshold is higher due to the shunting of currents to local return electrodes, which decreases the distribution of the fields through the retina away from the plane of the electrode array [16]. This trend is significant at electrode-to-cell distances greater than the electrode size [16]. The relatively higher activation threshold with the hexagonal configuration raises concerns about the efficiency of this mode of stimulation. Safe charge delivery and recovery necessitates a lower activation threshold than the safe charge injection limit of the electrode [17], implying that high threshold electrode configurations may not be applicable in neuroprostheses. In terms of power efficiency, activation threshold is regarded as the decisive factor determining the power consumption of the retinal neuroprosthesis [18], [19]. Therefore, the MP stimulation mode with a relatively lower activation threshold is superior in terms of safe and efficient stimulation.

Despite a lower activation threshold with MP stimulation, physiological studies in cochlear implants have argued that a smaller perceptual dynamic range may be obtained with MP stimulation, with a larger perceptual dynamic range achieved with a tripolar configuration, consisting of one active electrode flanked by two local returns [20]–[22]. However, the rate of increase of perception with increasing stimulating current depends on the electrode configuration, which is lowest with the tripolar configuration [19], [23]. Therefore, the threshold in cochlear implants under tripolar stimulation is higher than the MP configuration, and it may exceed safe charge injection limits [20]. Accordingly, if the safe charge limit is lower than the neural response saturation limit, a high-acuity hexagonal stimulation mode can only provide a narrow dynamic range. This drawback can theoretically be limited by using an electrode material with a higher safe limit. Alternatively, reducing the activation threshold appears to increase the dynamic range. Such a tuning scheme for dynamic range has not been evaluated in retinal implants. In retinal neuroprostheses, the perceptual dynamic range refers to the brightness dynamic range – the range from the darkest to the brightest phosphenes – of artificial visual perception. The brightness level of the perceived pixels is partly modulated by the stimulation intensity [24]. The amplitude modulation in an image processor for a retinal implant maps the wide dynamic range of brightness into the smaller electrical dynamic range available on the multielectrode array. This compression algorithm necessitates down sampling in the brightness domain. Accordingly, to optimally map the brightness dynamic range onto the dynamic range of stimulation, a wide dynamic range is needed.

Previous simulations predicted that stimulation with a hybrid mix of MP and multipolar configurations – whereby a fraction of current is returned through local return electrodes, and the remaining current flows through a distant return – could combine the advantages of both configurations by tuning the spatial field distribution [13], [21], [23], [25], [26]. Accordingly, a mix of hexagonal and MP configurations – quasi monopolar (QMP) – might be a compromise between low-threshold broad MP and high-threshold focused hexagonal configurations. This approach was recently proposed in a patent application by Suaning et al. [27] has assessed the effect of QMP on the activation threshold.

The present modeling study was carried out to compare the functional characteristics of QMP with the MP and hexagonal configurations. The potential for the QMP configuration to achieve a trade-off between the constructive and destructive effects of crosstalk was evaluated. In addition, the performance of the device across various sites of implantation – which defines the distance between the multielectrode array and the target cell layer – and various electrode geometry parameters were simulated. Electrode parameters included the number of electrodes, electrode size, and center-to-center electrode spacing. The results of this study can guide designs to improve the functional performance of retinal implants by using features of electrical crosstalk to maximal advantage.

## Methods

### Electric field model

**Figure 1:**
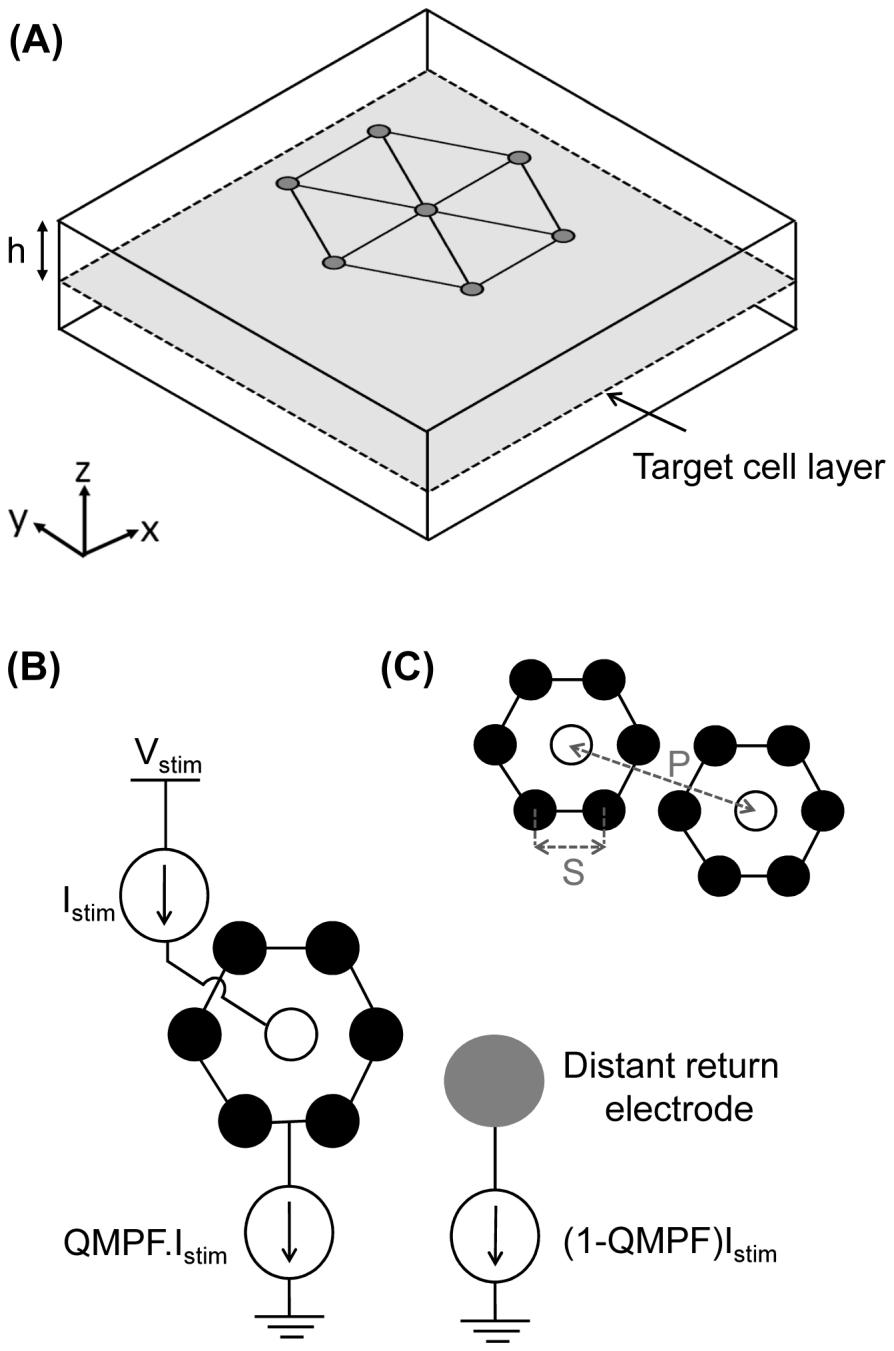
Layout of three-dimensional finite element model and electrode configurations. (A) Triangular lattice multielectrode array located on the upper boundary of 3D rectangular domain at a distance of *h* from the target cell layer. (B) Illustration of QMP configuration. The white (open) circle indicates an active electrode guarded by six local return electrodes (solid black circles). *I*_Stim_ is the magnitude of current at the active electrode, and *V_Stim_* is the voltage supply of the stimulator. The local return electrodes carry a fraction of the return current, defined by the QMP fraction (QMPF), with the remaining current delivered to the extra-retinal electrode. (C) Electrode spacing and center-to-center electrode pixel spacing are represented by *S* and *P*, respectively.

Performance assessment of the QMP configuration in a visual prosthesis requires an understanding of how the electric fields are distributed throughout the retina. A three-dimensional (3D) finite element model, consisting of an 8×8×0.5 mm^3^ rectangular domain, was employed to estimate the electric field intensity for a retina modeled as a passive homogenous conductive medium. The size of the medium relative to the array was large enough to avoid edge effects on the spatial field distribution. A schematic of the model is shown in figure 1A, where the multielectrode array was positioned on the upper surface of the passive domain at a distance *h* from the target cell layer. Various *h* distances from 20 to 400 µm were simulated to evaluate the effect of distance from the target retinal cells – in this case the RGCs and the multielectrode array – on the distribution of electric fields. In this simplified model the *h* distance therefore corresponds to a combination of implantation site – for example, sub-retinal versus suprachoroidal placement – as well as retinal thickness.

The model enabled the analysis of the functional performance of a triangular lattice multielectrode array. QMP is similar to the hexagonal configuration, except that the six disk-shaped return electrodes carry a fraction of the return current, while the remaining current is sent to the distant ground electrode (figure 1B). This fraction is defined as the QMP fraction (QMPF), and is equal to one for the MP mode where all the return current is pulled from the extra-retinal ground electrode, and is equal to zero for the hexagonal electrode configuration. In this study, the QMP configuration by convention refers to electrode configurations having QMPF between zero and one. The present study assessed variation of artificial visual acuity, threshold, dynamic range, and power consumption for the three electrode configurations – MP, QMP and hexagonal.

Besides the return path configuration, other electrode parameters are believed to contribute to variability in the functional performance of a given multielectrode array. Electrode diameters (*D*) of 50 and 100 µm were studied, to compare the simulation results with the clinical results reported by Zrenner et al. [2]. The center-to-center distance between the electrodes (*S*) ranged between 1000 µm and either 55 µm, for an electrode diameter of 50 µm, or 110 µm for diameters of 100 µm. Center-to-center pixel spacing (*P*) – where each pixel is defined by the location of an active electrode – equals *S* for the MP and 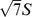
(figure 1C) for the hexagonal and QMP configurations.

The following Poisson equation was used to determine the distribution of the electric field (**E**) in V/m throughout the 3D retinal domain: 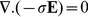
(1)


Where 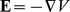
and σ is the isotropic conductivity of the homogenous conductive, taken as the conductivity of physiological saline (1.25 S/m) [28].

Imposed boundary conditions on equation (1) were that the extra-retinal ground electrode – the lower surface of the conductive medium – was constrained to zero volts. The product of the tissue conductivity (σ) and the derivative of the electric potential normal to the other boundaries was equal to the prescribed current density (*I_stim_*/*Area*) at all active electrodes, and equal to zero at all other boundaries – to simulate an insulating (zero-flux) condition.

A multi-resolution tetrahedral finite element mesh with mean element size of 7 µm was generated with the mesh refined on the boundary of the electrodes and along the z-axis to improve the accuracy of the solution. The meshed model was solved with COMSOL Multiphysics finite element software (v 4.1, COMSOL AB, Sweden).

### Threshold current

Threshold current was defined as the minimum current level needed to elicit an action potential in the target RGCs. Previous modeling studies of retinal stimulation [29]–[31] have used a Hodgkin–Huxley–type ionic model to estimate the current level to elicit action potentials in the RGCs. However, this approach is computationally-intensive. In the present study, an alternative method was employed to estimate the threshold current, based on a threshold level (|**E**|_th_) at a site corresponding to the RGC layer on the epiretinal surface. A previous modeling study estimated |**E**|_th_ for chronaxie pulse durations by converting the stimulus current strength-duration curve into an |**E**|_th_ – duration curve [32]. The present study estimated electrode current threshold by constraining the magnitude of the electrode field (evaluated from all components in 3D) to an |**E**|_th_ value of 1116 V/m at desired distances from the array, as determined by the former study [32].

### Adjacency effect

During parallel stimulation, the activation threshold of each electrode is influenced by crosstalk. A determining factor for crosstalk is the number of active neighboring electrodes surrounding each electrode. In the present study, this quantity is referred to as adjacency. The effect of adjacency on crosstalk was quantified by the threshold shift, defined as the difference in threshold between single and multielectrode stimulation. A larger shift indicated a greater facilitatory effect of crosstalk.

**Figure 2:**
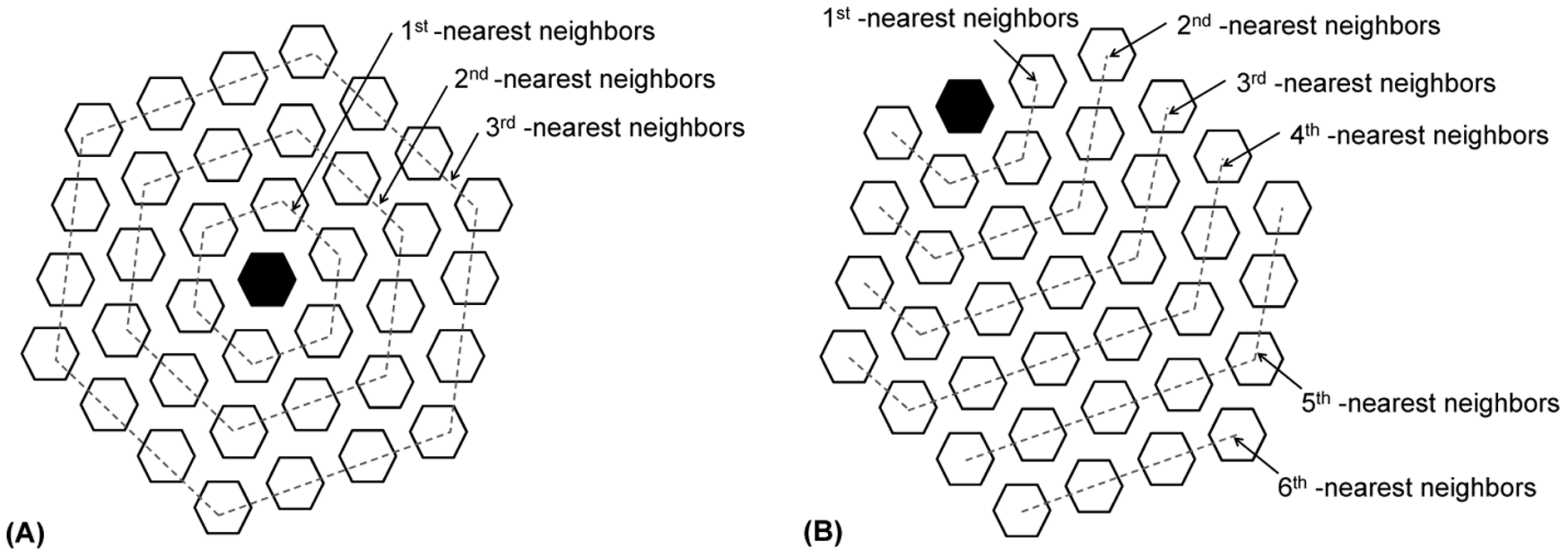
Schematic of *N^th^*-nearest neighbors of the central electrode pixel of the array (A) and an electrode pixel on the boundary (B). The relevant electrodes are represented by filled hexagons. Each hexagon (open and filled) represents a group of seven disk-shaped electrodes: an active electrode in the center of the hexagon with six guards each occupying one of the vertices.

The adjacency effect on crosstalk is in turn related to the distance between electrodes, namely the pitch of the multielectrode array. Accordingly, we would expect that the effect of adjacency is more exaggerated for the nearest neighbors. Therefore, neighboring electrodes can be divided into *N* adjacency subgroups based on the distance *d* from the electrode. Each subgroup encompasses the *N* ^th^-nearest neighbors having equal *d*. In the example shown in figure 2, the neighbors of the central hex of the array can be grouped into three categories: first, second and third nearest neighbors, while there are six subgroups for the outer electrode on the boundary of the array.

The dependence of the adjacency effect on the position of the electrode in the array leads to a variation in threshold across all electrodes of the array. It is likely that the sensitivity of the array to adjacency is partly a function of the electrode configuration. Since electrode-to-electrode variability is important for stimulus programming of the device, it is essential to understand the adjacency effect across the electrodes of the array. Therefore, standard deviation of electrode threshold for all electrodes in a given configuration was determined.

### Artificial visual acuity

The visual acuity of artificial vision is defined by the spatial sampling frequency of electrode stimulation having a high contrast on/off grating pattern. Artificial visual acuity has therefore two aspects: spatial sampling frequency and contrast. Normal spatial sampling frequency in the human is defined as the ability to resolve a spatial frequency of 1 minute of arc, which is equal to 1/60 of a degree [33]. Since one degree of visual angle covers ∼280 µm of the retina, the maximum spatial sampling frequency is 280 (µm)/60 (cycl). Accordingly, the spatial sampling frequency for retinal implants in decimal notation is [8]: 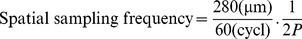
(2)where *P* denotes the center-to-center pixel spacing. In the present study, the artificial visual acuity is expressed in Snellen notation: 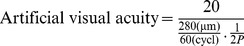
(3)Contrast refers to the ratio between the difference in magnitude of the electric field between stimulated and non-stimulated areas of a one-dimensional grating stimulation pattern and the mean magnitude of the electric field: 
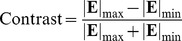
(4)where |**E**|_max_ and |**E**|_min_ denote the maximum and minimum magnitudes of the electric field. The contrast for grating stimulation was estimated at the threshold for each electrode configuration. This study determined artificial visual acuity using a modeling framework equivalent to that of Wilke et al. [5].

### Dynamic range

Dynamic range is often defined as the difference between the threshold level stimulation and the comfortable level stimulation. However, a clinical study in cochlear implants has shown that the comfortable level of stimulation for a focused electrode configuration is not achievable within the safe charge injection limit and thus the safe charge injection limit is the upper limit for the stimulation [20]. In the present study, the dynamic range of a retinal prosthesis is defined as the difference between the charges required to elicit minimum perceptible and safe maximum brightness levels. The minimum detectable level of brightness is equivalent to the threshold of activation, whereas the maximum brightness level is that which can be achieved at the charge injection limit for the electrode material. We define the dynamic range as the difference in decibels between a given threshold of charge density (*ρ_th_*) and the safe charge injection limit (*ρ_s_*): 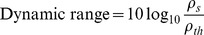
(5)


To calculate the threshold charge, the threshold current was divided by a pulse width equal to the chronaxie obtained in the study of Sekirnjak et al. [12], namely 407 µs. Maximum safe charge injection limits of 0.2 mC/cm^2^ for platinum [34] and 1–9 mC/cm^2^ [35] for sputtered iridium oxide film (SIROF), were assumed.

### Power consumption

Although the ideal electrode design for a high-acuity retinal implant necessitates crosstalk elimination, crosstalk will have the beneficial effect of reducing device power consumption by reducing the total sum of stimulus currents through all active electrodes of the array. The crosstalk reduction effect on power consumption was quantified by the percent reduction in power consumption relative to ideal multielectrode stimulation, in which there is no crosstalk between electrodes. We refer to this quantity as power conservation. With a chronaxie of 407 µs [12], the electric power of the device is directly related to the product of the root mean square (rms) of total stimulus current and stimulator voltage (*V_Stim_*) by Joule's law. Considering *V_Stim_* as a constant, the ratio of power consumption of a non-ideal to ideal device is a function of rms stimulus current. The power consumption of the device was calculated assuming that the stimulus current is the principle source of power consumption and also the electrode impedances remained constant.

## Results

### Electric field and potential profile

**Figure 3:**
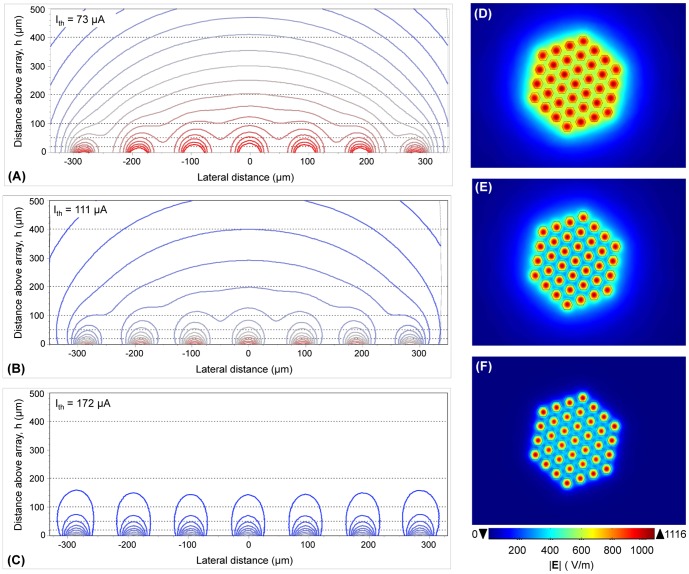
Simulated electric field in the *h* = 0 µm (A–C) and h = 100 µm (D–F) plane, as well as electric potential (G–I) in an xz-plane (see figure 1) during multielectrode stimulation for the following electrode configurations: MP (A, D and G), QMP at a QMPF of 0.5 (B, E and H), and hexagonal (C, F and I). The isopotential contours are equi-spaced, ranging from 0.05 to 0.7 V in steps of 0.01 V, with the higher potentials occurring in the plane of the electrode array. Threshold current, *I_th_*, is shown in the upper left corner of each panel. Electrode diameter and center-to-center electrode spacing were 50 µm and 55 µm, respectively. Note that the panels G-I are zoomed in views of the volume conductor domain.

The effect of electric crosstalk on the spatial distribution of electric potential evoked by simultaneous multielectrode stimulation was simulated for various electrode configurations: MP, hexagonal, and QMP with a QMPF of 0.5. The spatial spread of electric potential was compared at an electric field intensity of 1116 V/m at a distance of 100 µm beneath the central electrode of the array (figure 3A–I). The potential profiles were obtained from simultaneous stimulation of 37 electrodes with an electrode diameter of 50 µm and a center-to-center electrode spacing of 55 µm. The current level that is shown in the upper left corner of panels G-I was that required to reach an electric field magnitude of 1116 V/m – deemed here as the threshold field for target cell activation – at a depth of 100 µm.

The MP configuration resulted in the greatest spatial spread of potential in both the lateral and longitudinal directions. The lateral distribution is the result of the transverse electric field components, while the longitudinal spread is in the direction normal to the surface of the electrode, that is the z-axis. To decrease the stimulus threshold, greater longitudinal distributions are favorable; however, more lateral distributions result in a higher degree of crosstalk. From the results of figure 3, the QMP configuration elicits a more focused electric field pattern than the MP case, so the use of this mode of stimulation yields a consistently lower degree of electrode-electrode interference (crosstalk) at distances closer to the array than the electrode size (*D*). Unlike MP and QMP, there is no crosstalk effect on the narrow potential profiles induced by the hexagonal configuration up to distances greater than the electrode size from the array.

### Contrast

**Figure 4:**
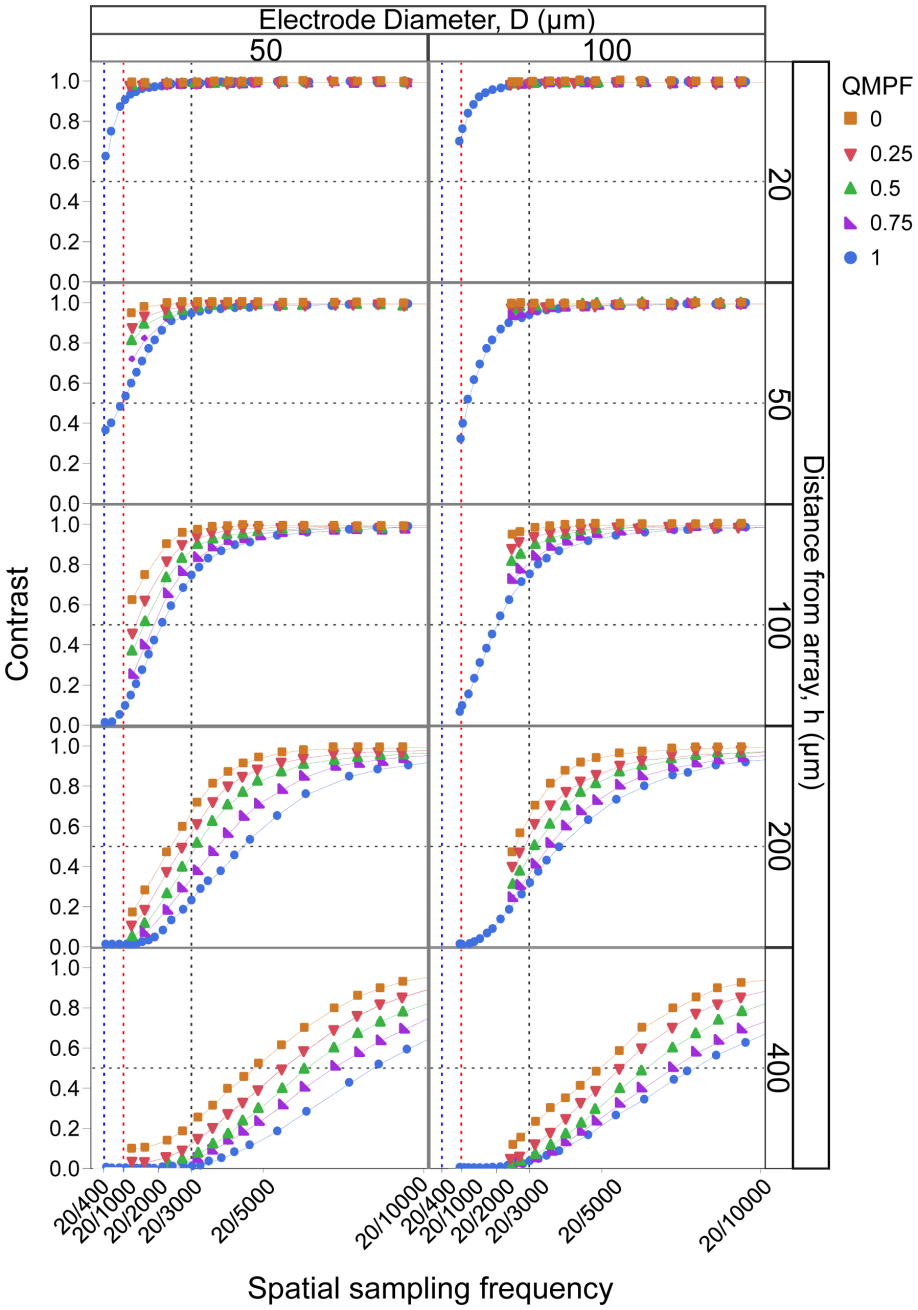
Contrast as a function of artificial visual acuity expressed in Snellen notation for various distances from the array. Electrode configuration varies from hexagonal to QMP and MP, as denoted by the QMPF. A QMPF of 1 is equivalent to the MP mode, and 0 to the hexagonal configuration. Legal blindness (20/400) is marked by a blue vertical line. The red vertical line indicates a artificial visual acuity of 20/1000. The horizontal dashed line represents the contrast threshold of 0.5.

The electric crosstalk contribution to contrast was determined across the range of electrode configurations and parameters. Figure 4 illustrates contrast as a function of artificial visual acuity for various distances from the array to the RGC layer (horizontal rows), where the symbols indicate the QMPF, and consequently the electrode configuration. The left and right columns show the variation in contrast for electrode diameters of 50 µm and 100 µm, respectively. The contrast monotonically decreases as a function of distance from the array. For each panel, contrast increased as the artificial visual acuity decreased. Contrast enhancement followed a sigmoidal relationship with spatial resolution. With a given artificial visual acuity, contrast decreased as the electrode configuration was changed from hexagonal to QMP to MP, represented as rightward shifts of the sigmoidal curves. The size of this shift was directly related to the degree of crosstalk. These results suggest that by restricting the size of the electric potential spread using local return electrodes, spatial interactions between parallel stimulated electrodes are greatly reduced. Among the QMP configurations, there was a consistent trend of increasing contrast with increasing QMPF. However, at distances smaller than the electrode dimensions, there was no significant difference in contrast between electrode configurations.

Utilizing a conventional contrast threshold of 0.5 [7] (the horizontal dashed line in figure 4), the results indicate the maximum possible artificial visual acuity able to reach this threshold. At distances smaller than the electrode size, the maximum artificial visual acuity was obtained using the MP configuration – which was 20/400 (legal blindness [7]), and 20/1000 (sufficient sampling frequency to resolve grating stimulation [2]) – with electrode diameters of 50 µm and 100 µm, respectively. In order to maintain artificial visual acuity at distances greater than the electrode dimensions, stimulation with the hexagonal configuration is most favorable. Far from the electrode array, the QMP configuration provides no advantage over either the MP or hexagonal configurations in terms of spatial resolution.

### Adjacency effect

**Figure 5:**
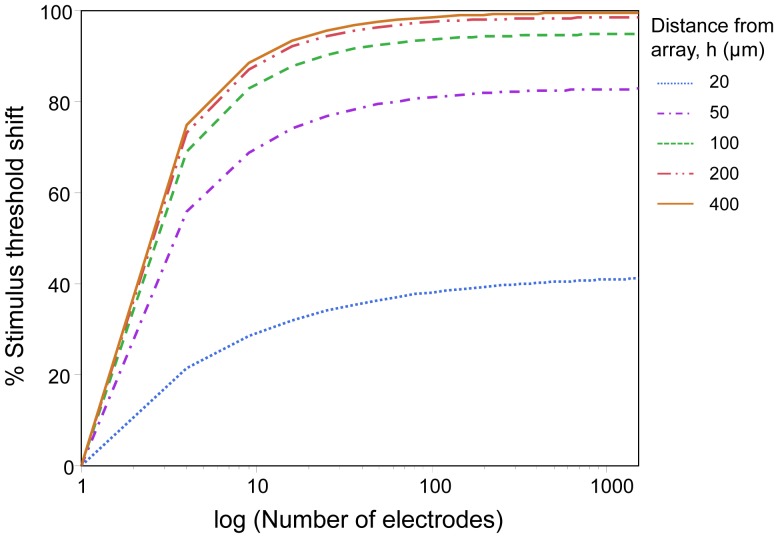
Threshold shift in percent as a function of adjacency for the central electrode of the array at various distances from the array. The electrode configuration was MP, with an electrode diameter of 100 µm and a center-to-center electrode spacing of 110 µm.

Figure 5 shows the dependency of crosstalk on electrode adjacency under parallel stimulation. The shift in threshold current for the central electrode in a multielectrode versus single electrode configuration was determined. Simulations were undertaken for the concurrent MP stimulation utilizing 1 to 1500 total electrodes. Electrode diameter (*D*) and the center-to-center electrode spacing (*S*) were 100 µm and 110 µm respectively. The results of figure 5 indicate that threshold shift increases with increasing adjacency. The growth rate of threshold shift was lower for closer distances to the array, while there was a slight difference between the rates at distances greater than the electrode size (100 µm). These results correspond to our previous findings [10]. Irrespective of the distance from the array, the threshold shift for the central electrode saturated when it was surrounded by 50 electrodes. This case described the general trend across various electrode configurations.

**Figure 6:**
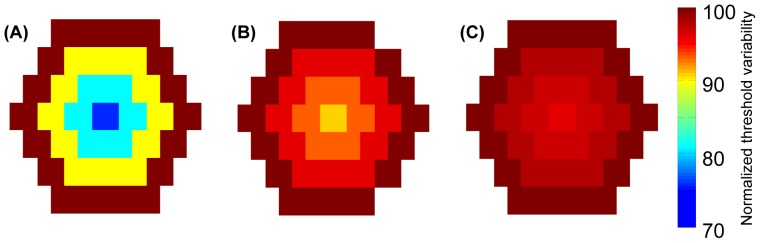
Electrode-to-electrode variability in stimulus threshold in the xy-plane at a distance of 100 µm from the array. Electrode diameter and center-to-center electrode spacing were 100 µm and 110 µm respectively, with the colorbar representing normalized threshold. The simulations shown are for MP (A), QMP at QMPF of 0.5 (B), and hexagonal (C) configurations.

To evaluate the sensitivity of threshold to the adjacency of its first, second and third neighborhood, the electrode-to-electrode variability in stimulus threshold was estimated. An example of the distribution of threshold across electrodes for an electrode diameter of 100 µm and a center-to-center spacing of 110 µm is illustrated in figure 6. Each panel represents data in the xy-plane at a distance of 100 µm from the array for each electrode configuration: MP (figure 6A), QMPF of 0.5 (figure 6B), and hexagonal (figure 6C). Since each electrode configuration spans a different range of thresholds, these thresholds were normalized to within the range of 0 and 100, where 100 was assigned the maximum threshold in each electrode configuration to facilitate comparison across configurations. The maximum threshold was observed for electrodes lying on the outer boundaries, which have the lowest adjacency.

With the hexagonal configuration, the thresholds were relatively identical for all electrodes. However, the threshold for those electrodes closest to the center electrode was slightly lower due to field summation. This trend is progressively more significant for the QMP and MP configurations. Accordingly, the MP mode is the most sensitive configuration with respect to the number of electrodes in adjacent neighborhoods, in accordance with the broad spread of the electric field with this configuration.

**Figure 7:**
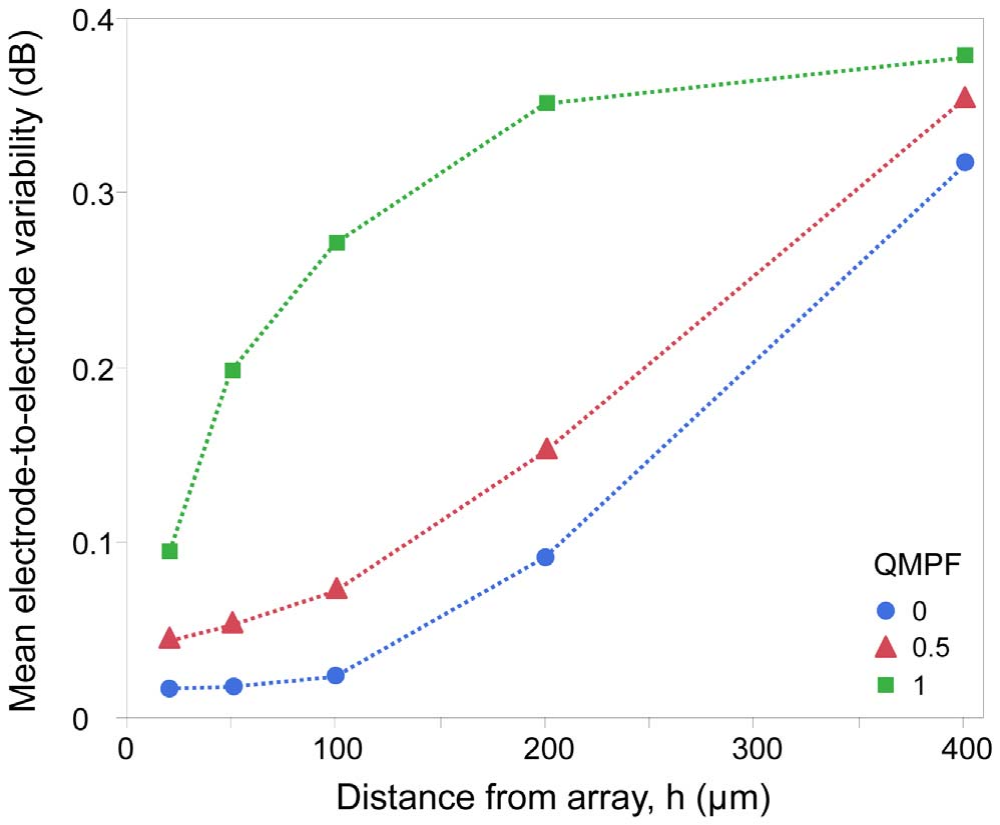
Distribution of electrode-to-electrode variability. The symbols represent the QMPF, and hence, the electrode configuration. A QMPF of 0 and 1 are equivalent to the hexagonal and MP electrode configurations, respectively.

The mean difference between stimulus thresholds of each electrode for each of the above arrays as a function of distance from the array was determined. Consistent with the case shown in figure 7, when comparisons were made for all distances from the array, the greatest electrode-to-electrode variability occurred for the MP configuration. The mean electrode-to-electrode variability increased more rapidly as a function of distance for the array with the MP mode than the two other configurations. This difference is likely a result of crosstalk at distances from the array less than the electrode size with the MP configuration, and greater than the electrode size with the QMP and hexagonal configurations, consistent with the results of figure 3.

### Dynamic range

**Figure 8:**
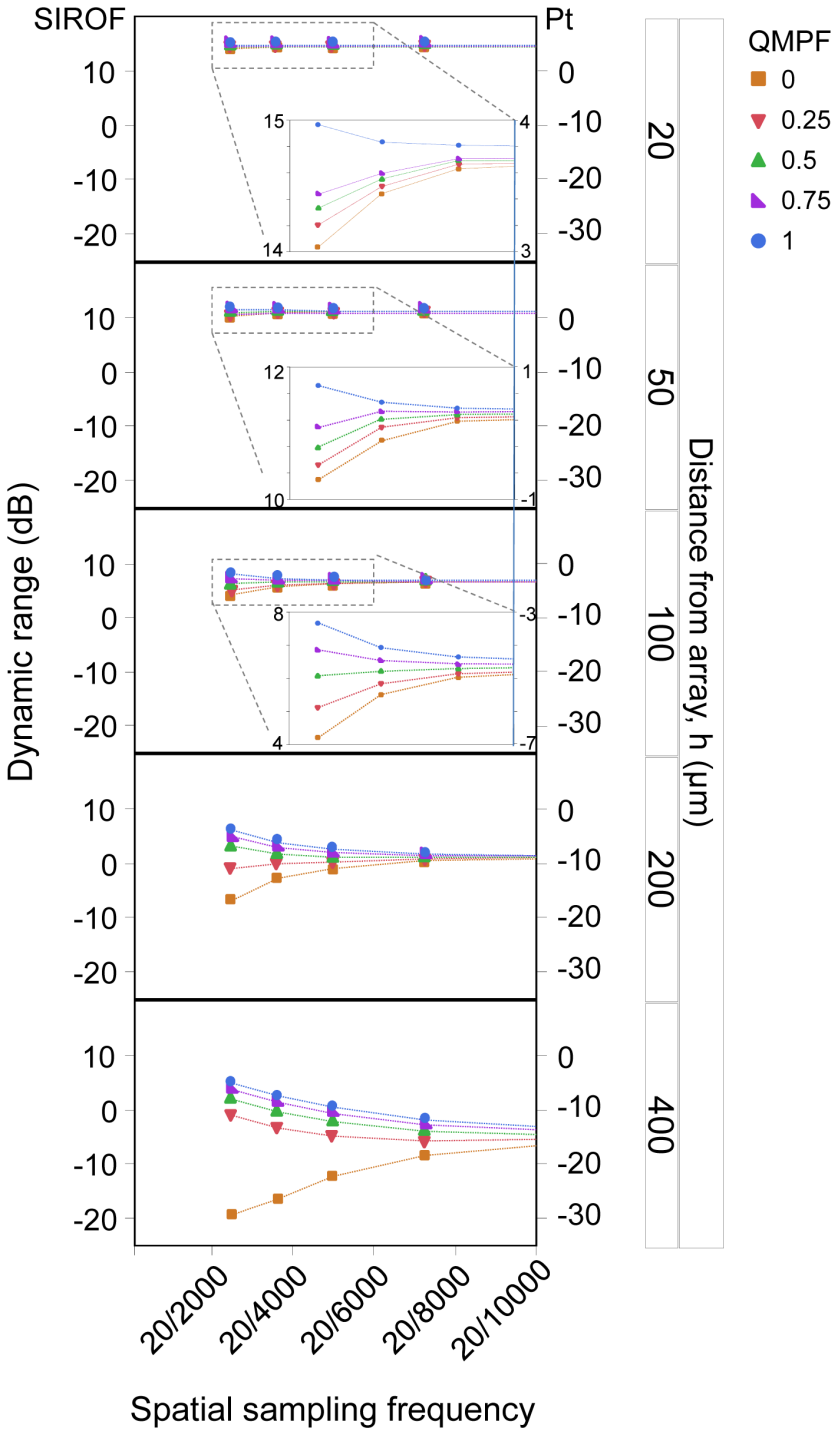
Dynamic range as a function of artificial visual acuity for electrode materials of SIROF (the left ordinate axis), and platinum (the right ordinate axis). Each panel represents the dynamic range for a given distance from the array for various electrode configurations.

A major goal in designing multielectrode arrays for retinal implants is to develop a wide dynamic range device. In this study, dynamic range refers to the difference between the threshold and the safe charge injection limit. Our simulations demonstrated variation in dynamic range across electrode arrays using SIROF and platinum electrodes. The disparity in dynamic range between electrode configurations as a function of artificial visual acuity is shown in figure 8 for an electrode diameter of 100 µm. A safe charge injection limit of 2 mC/cm^2^ was used for this electrode size [35]. Each row in figure 8 plots the threshold at a fixed distance from the array, with data symbols representing different electrode configurations, as in figure 4. The left and right vertical axes denote dynamic ranges for SIROF and platinum, respectively. The points above 0 dB, which represents the safe stimulation limit, indicate the electrode configurations applicable for safe stimulation with the corresponding material.

In general, the dynamic range was constant across the electrode configurations and tended to be larger at closer distances to the array. Since the safe charge injection limit was a fixed material property, the dynamic range was strongly dependent on the stimulus threshold. Accordingly, a large dynamic range can be obtained with low threshold configurations. In each panel shown in figure 8, the dynamic range decreased as the configuration changed from MP to QMP to hexagonal, consistent with the expected decrease in electric field penetration depth. These simulations show that with the hexagonal configuration, the dynamic range increased as a consequence of the decreasing artificial visual acuity. This tendency was due to penetration depth enhancement with an increase in electrode spacing. With the MP configuration, a decrease in the dynamic range as a function of decreases in the artificial visual acuity was due to crosstalk reduction, which ameliorated the summation effect of each electrode. At distances smaller than the electrode size, the QMP configuration followed a trend similar to the hexagonal configuration, whereas at distances greater than the electrode size, there was a tendency for QMP to be similar to the MP configuration. With a QMP of 0.5, the threshold was relatively constant at a distance from the array equal to the electrode diameter. One possible explanation is that this configuration is a trade-off between the reducing effect of crosstalk and the increasing effect of penetration depth.

For target cells at distances up to the electrode dimension, both platinum and SIROF can provide a positive dynamic range, corresponding to safe stimulation. However, the safe stimulation of more distant target cells was only obtained with SIROF for both the MP and QMP configurations. The superiority of relatively focused QMP stimulation over the hexagonal configuration with respect to safe stimulation is significant at distances greater than the electrode diameter, whereas the hexagonal configuration is not applicable at all. A similar trend was observed for an electrode diameter of 50 µm.

### Power consumption

**Figure 9:**
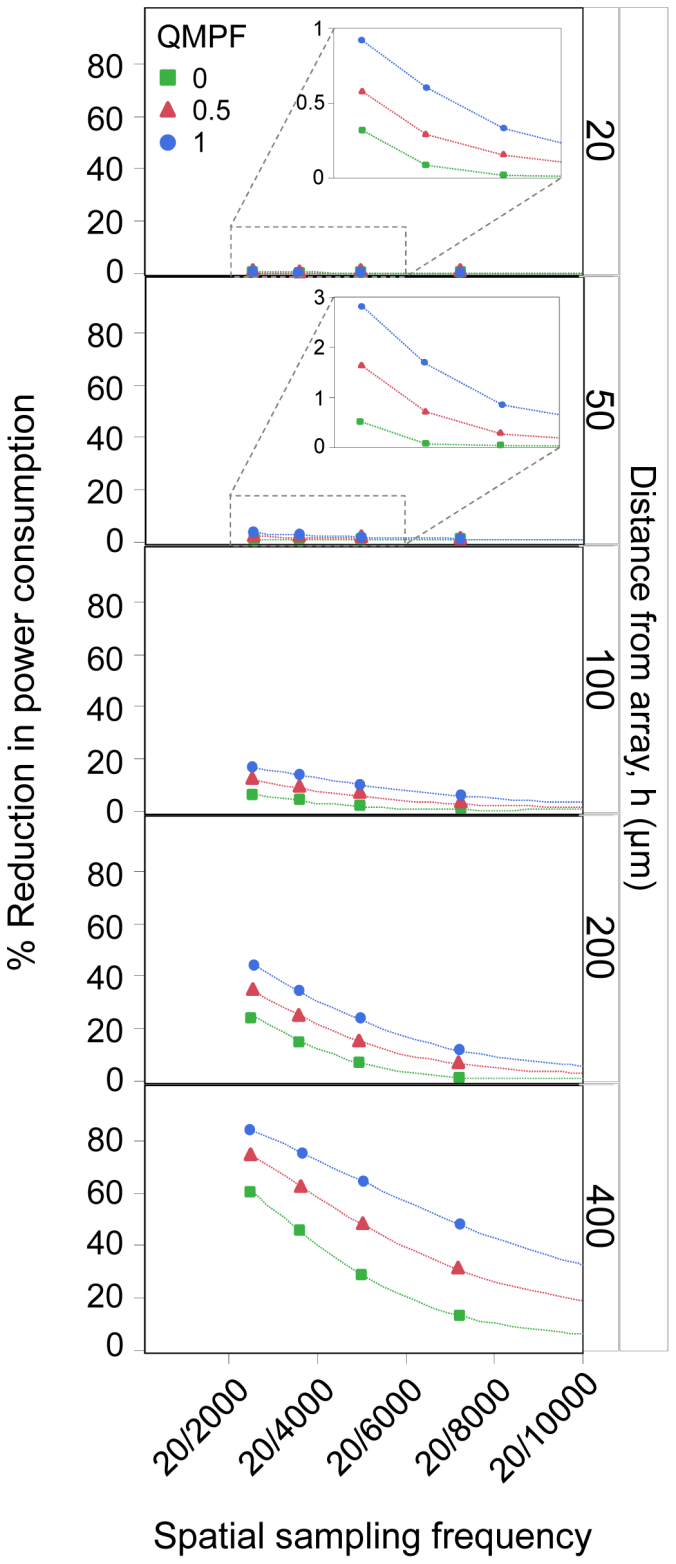
Decrease in power consumption for various electrode configurations and distances from the array. Each panel represents a decrease in the power consumption as a function of artificial visual acuity.

A reduction in the power consumption of the device relative to the ideal electrode configuration is illustrated in figure 9, in order to quantify the contribution of crosstalk to power consumption. Each panel of this figure shows a condition in which the distance to the array was held constant, and the artificial visual acuity was decreased by increasing the center-to-center pixel spacing. The various symbols represent MP, QMP with QMPF of 0.5, and hexagonal electrode configurations. With all electrode configurations, the relative power conservation increased from a distance of 20 µm to 400 µm, consistent with the presumed increase in crosstalk.

The reduction in power consumption increases at further distances from the array. At a fixed distance, reducing the artificial visual acuity helps to decrease power conservation, which leads to a low-efficiency device. As expected, the simulation results demonstrated the trend that power conservation was highest under MP stimulation, and lowest for the hexagonal configuration. The higher level of power conservation with QMPF <1 has the advantage of saving battery life over the hexagonal configuration. Power consumption progressively increases from the highest to the lowest QMPF, regardless of artificial visual acuity.

## Discussion

In the present study, the ability of the QMP configuration to make a trade-off between positive and negative effects of crosstalk was compared against the MP and hexagonal array electrode configurations.

To compare the destructive interference of crosstalk across the electrode configurations, the spatial distribution of the induced electric fields was solved for over a range of electrode configurations. Comparing the effect of QMP and hexagonal configurations on field distribution, our simulations indicated that QMP leads to a loss of spatial selectivity compared to the hexagonal configuration, and consequently impairs artificial visual acuity. Nonetheless, the effect of crosstalk on visual acuity with QMP stimulation was less than that of MP. This trend of decreasing contrast from MP to QMP to hexagonal configurations is in agreement with previous studies which estimated activation patterns for MP, tripolar and partial tripolar configurations [23], [25].

The contrast of a grating stimulation pattern can be improved by increasing the distance between pixels (pitch), and thus decreasing the sampling frequency of the device. This trend was observed with all electrode configurations. However, at distances from the array up to the electrode dimension, the MP configuration provided a contrast threshold of 0.5 [8] with higher artificial visual acuity. Therefore, the potential benefit of employing other configurations can be justified only for distances from the array greater than the electrode sizes. Accordingly, it is unlikely that the QMP and hexagonal stimulation modes possess an advantage over MP stimulation for electrode diameters greater than the retinal thickness (∼150–200 µm for a human retina with advanced stages of degeneration as in retinitis pigmentosa [8]). However, Sekirnjak et al. suggested that an electrode diameter of 10 µm to 15 µm might be a compromise between spatial selectivity and safety, thus constituting the ideal electrode size for retinal implants [12]. With this electrode size, the QMP and hexagonal configurations are superior to the MP mode, regardless of their implantation site, though QMP with a lower activation threshold might be preferable.

Although the QMP configuration has the advantage over the hexagonal mode in terms of threshold and lower sensitivity to retinal remodeling, electrode-to-electrode variability in the stimulus threshold indicated that crosstalk under QMP was more sensitive to the position of the electrode array. This variability in threshold leads to variation in the dynamic range across the electrodes of the array. Accordingly, each electrode needs to be controlled independently, resulting in complex programming of the array in the clinical setting. It should be noted that this drawback strongly depends on the ratio of electrode size to the distance between the array and the target cell layer, with higher sensitivity occurring at small distances from the array less than the electrode size. Another disadvantage of an uneven distribution of threshold across electrodes is that the dynamic range of the outer electrodes of the array – where they have less adjacency – is lower than that of the central electrodes. For each electrode in the array, the dynamic range of stimulation is partly related to the number of brightness levels of the corresponding pixel of perception [24]. Therefore, the capability of brightness discrimination is somewhat limited for the outer electrodes, leading to possible aberrations in brightness-to-stimulation mapping.

The dependency of activation threshold on the location of the electrode for the QMP configuration can be reduced by increasing the center-to-center pixel spacing, and thus decreasing crosstalk. Reducing artificial visual acuity results in improved contrast, due to reduced crosstalk, which leads to an increase in power consumption in the array. This implies that an array with low artificial visual acuity is less efficient in increasing the life of batteries that power a retinal implant, for a fixed number of electrodes. Also, power consumption is directly related to the potential thermal insult that the device may induce in the retina [36] due to increased temperatures at the implant surface [5]. Palanker et al. [5] assumed a temperature elevation of 1°C as the safe limit, and calculated the maximum limit of power consumption, thus setting the maximum limit on the pixel density of the array. However, their study did not include the effect of crosstalk on power consumption. The present study elucidated the constructive effect of crosstalk on power, suggesting that high pixel densities with high degree of crosstalk – and eventually lower power conservation – are desirable for safe chronic stimulation of the retina. For high pixel densities, it has been proposed that an electrode material with higher safe charge injection limits than platinum, such as SIROF, should be employed [37]. Stimulation using electrodes made from SIROF will also affect the dynamic range of stimulation.

Employing an electrode material with a higher safe charge injection limit is one approach for increasing the dynamic range, though the advantage of employing SIROF over platinum is only significant at array distances of twice the electrode diameters for the MP and QMP configurations. In order to improve the dynamic range beyond this distance, a material with a higher safe limit than platinum might be preferable. The main challenge for replacing conventional platinum electrodes with new materials having a higher safety charge injection limit, such as SIROF and titanium nitride, is that these materials are not well established for chronic implantation. Therefore, the approach of increasing the dynamic range by optimizing the electrode configuration using well-established and tested electrode material such as platinum is more convenient and advantageous than employing a new electrode material.

Simulations of the dynamic range across electrode configurations demonstrated that improved intensity discrimination is obtained for QMP compared to the hexagonal stimulation mode. Further enhancement in the dynamic range might be possible with MP. At a given artificial visual acuity, there are two key factors which can provide a higher dynamic range with broadly distributed electric field configurations. The first is a greater penetration depth, as has been noted in previous studies [20], [23], [26]. The second is related to the reducing effect of crosstalk on the threshold. The superposition of electric field profiles for individual electrodes is more significant with broadly-distributed electric field modes. As a result, the superiority of broadly-distributed configurations compared to the hexagonal mode is more pronounced at high spatial frequencies, where the penetration depth of the hexagonal configuration is limited, and even more exaggerated at distances from the array greater than twice the electrode diameter, where the crosstalk effect is more significant.

When the center-to-center pixel spacing is increased, the dynamic range with the broad-spread configurations decreases, whereas it increases with the focused hexagonal mode. The reason is that the activation threshold with MP and QMP increases due to the decrease of the current–summation effect, thereby decreasing the dynamic range. However, the increase in penetration depth with increasing pixel spacing, as opposed to crosstalk reduction, is the dominant factor underlying the hexagonal mode, which results in a lower threshold and eventually a higher dynamic range for low spatial frequencies. Nevertheless, the dynamic range for the hexagonal configuration is still lower than that of QMP. The difference in dynamic range strongly depends on QMPF: increasing the QMPF allows a device with a lower threshold to enhance its intensity discrimination.

The QMPF also affects the response of QMP to increases in pixel spacing. For a QMPF of 0.5, pixel spacing has no significant effect on the dynamic range at a distance from the array equal to the electrode diameter (figure 8, third panel). This result suggests that the threshold at a QMPF of 0.5 is independent of pixel separation, which results in a constant power consumption. Therefore, pixel spacing can be used as a factor for optimizing the encoding of visual information in a visual neuroprosthesis. Increasing the pixel spacing in a given region of the array can improve the contrast of perception and convey more edge information. The accurate encoding of edge information is a matter of concern, as human vision perception operated principally on the edge information of an object [38].

The present study evaluated the dependency of constructive and destructive interferences of crosstalk on electrode array design in retinal implants. A previous modeling study that examined the activation patterns for partial tripolar configurations suggested that focused electrode configurations are capable of eliciting selective activation patterns even at suprathreshold levels [23]. However, that study assessed the ability of focused activation for single electrode stimulation only. There has yet to be a clear description of the effect of crosstalk on spatial selectivity at suprathreshold current levels.

The crosstalk is mainly considered as a drawback of parallel stimulation of the array. Experimental results in cochlear implants have revealed that the spatial interaction of electrical fields can be reduced by sequential stimulation of adjacent electrodes [39]. Evaluating the efficiency of this approach in crosstalk reduction requires an active model of the RGCs to simulate properly. In other words, the interleaving exploits the time-dependent properties of target cell activation, however, the present model is a passive simplification of activation.

Finally, studies on a hybrid multielectrode array should also consider the time-dependent effects of stimulating current on crosstalk, and its dependence on electrode design. Frequency is known to be a factor affecting psychophysical responses in retinal implants [5], [40]. Therefore, it is essential to optimize the electrode design for a sufficient stimulation frequency to maintain the spatiotemporal resolution of artificial perception. In future modeling work, a combination of the present approach with an active model of the retina [29] needs to be undertaken. Such a model will need to be experimentally validated in order to make reliable predictions on the performance of a future retinal prosthesis for clinical implications.

### Model limitations

The model described in this study was developed to compare the essential functional characteristics of various electrode configurations in a retinal prosthesis. The model did not include detailed anatomical and physiological properties of the retina, which would be useful in accurately simulating the temporo-spatial patterns of retinal activation. For example, it is known that there are several different classes of retinal ganglion cells in the primate retina, with differences in morphological structure (e.g. cell size, dendritic field), network connectivity, as well as intrinsic electrophysiological properties [41]. Such a variation will likely lead to some spatial heterogeneity in threshold, however, such a detailed active model of retinal activation was beyond the scope of the present study. Yet, the methods of this study could be extended in future to incorporate active models of RGCs in the target layer.

The main limitation in the accuracy of this modeling study is that the implemented electric field threshold has not been obtained experimentally, but inferred from existing literature [32]. An alternate way of ascertaining activation in the target cell layer is to use an activating function, whereby axonal activation is deemed to take place if the second derivative of extracellular potential in the direction of the axons exceeds a given threshold [30], [42]. This method, however, requires knowledge of the axonal orientation at every point in the target layer and more importantly, assumes we seek to activate the axons directly as opposed to the RGC somas or initial segments. The latter is desirable, since it is well established that direct axonal activation leads to undesirable smearing and elongation of phosphenes. For non-axonal substructures such as the soma or initial segment, a generalized activation function can also be implemented based on a compartmental description of the neuron [42], but again, this requires knowledge of the location of these substructures relative to each other and to the applied field orientation. The method used in this study represents a simpler approach, and is valid to the extent that the same electric field threshold value was used to compare the functional performance of different electrode designs.

In addition, our model assumes a homogeneous medium with low electric resistance akin to that of physiological saline. In reality, the retina is composed of various layers of differing conductivity which should be taken into account in future modeling studies. At present however, these conductivities have not been ascertained for the human retina, and this study represents only a first step in understanding retinal stimulation in a vision prosthesis multi-electrode array under quasi-monopolar stimulation.

Despite the simplifications made in the present study, the findings are in strong agreement with existing modeling and clinical studies in cochlear implants, which highlight the predictive capability of similar models [20]–[22], [25], [26]. In future, the model described in this study could incorporate additional features to address different levels of complexity, including detailed and complex characteristics of the electrode–retina interface, as well as the biophysical and anatomical properties of the retina. The main advantage of incorporating active neural models of the retina is the evaluation of time-dependent stimulation characteristics.
